# Cognitive impairment in hereditary spherocytosis

**DOI:** 10.1111/bjh.70088

**Published:** 2025-08-15

**Authors:** Immacolata Tartaglione, Maria Grazia Paturzo, Serena Picazio, Cristina Malvone, Delia De Biasio, Domenico Roberti, Federica Di Gennaro, Marcella Contieri, Giovanni Librizzi, Maria Agnese Pirozzi, Fabrizio Esposito, Silverio Perrotta, Renzo Manara

**Affiliations:** ^1^ Dipartimento della Donna, del Bambino e di Chirurgia Generale e Specialistica Università degli Studi della Campania “Luigi Vanvitelli” Napoli Italy; ^2^ Neuroradiologia Università degli Studi di Padova Padova Italy; ^3^ Dipartimento di Scienze Mediche e Chirurgiche Avanzate Università degli Studi della Campania “Luigi Vanvitelli” Napoli Italy

**Keywords:** anaemia, cognitive impairment, spherocytosis

To the Editor,

There is growing evidence that chronic haemolytic anaemias may present with lower cognitive performances both in children and in adults in spite of best clinical practice. Functional impairment might be unnoted even by experienced clinicians but might impact on the quality of life and is revealed by cognitive testing. Most data refer to sickle cell anemia^1^ and beta‐thalassaemia,^2^ though cognitive deficits have been described also in specific subgroups of Blackfan–Diamond anaemia^3^ and alpha‐thalassaemia.^4^ The pathogenesis of brain impairment is elusive, probably because causative factors are multiple and likely vary among different anaemias. Strokes and silent vascular‐like white matter changes, for example, have shown a causative relationship in sickle cell anemia^5^ while their role in other haemolytic anaemias seems to be absent or at least questionable.^6^ In some conditions, genetics seem to be prominent as cognitive impairment is present in specific mutations that lead to abnormal myelination or early atrophy^4^; in other anaemias, conventional and advanced magnetic resonance imaging (MRI) investigations do not show signs of brain involvement in spite of documented functional impairment.^2^, ^6^ In these patients, other factors such as frequent hospitalization since early ages, decreased life expectations and comorbidities might exert some effects on brain development and functioning, rendering the identification of causative factors rather complex. To make this picture more complex, some confounding factors are sometimes endorsed even in contrast with literature evidence. In beta‐thalassaemia, for example, some authors^7^, ^8^ insist in claiming a role of brain iron overload even though MRI studies^9^ and, above all, a pathological study^10^ have denied any increase of iron in the neural tissue and showed no relationship with cognitive impairment.^9^

So far, no study has investigated cognitive functioning in hereditary spherocytosis (HS, Minkowski–Chauffard disease, #182900), a disease characterized by chronic haemolytic anaemia with splenomegaly, jaundice and spherocytes in peripheral blood films.^11^ The most severe forms of HS require transfusions since early childhood for preventing multi‐organ failure. In these patients, splenectomy is an effective therapeutic option, as it may result in transfusion independence. However, splenectomy may favour a hypercoagulative state with an overall increased risk of thrombosis^12^, ^13^ even though adult HS patients do not seem to show increased brain parenchymal vascular lesions compared to age‐matched controls (unpublished data).

We studied 29 adult HS patients (mean age 34 ± 13.4 years, range 16–67 years; 13 females; mean haemoglobin (Hb) 13.5 ± 1.7 g/dL; platelets (PLT) 376  ± 149.8 x 10^9/L, total bilirubin 1.8 ± 1.3 mg/dL, lactate dehydrogenase (LDH) 258 ± 73.3 iu/mL; 20 splenectomized, mean age at splenectomy 11.5 ± 6.7 years; for the splenectomized group, mean Hb pre‐splenectomy was 9.3 ± 1.7 g/dL, with transfusions every 4.0 ± 3.4 months and pre‐transfusion Hb 5.4 ± 0.4 g/dL) by Wechsler Adult Intelligence Scale—Fourth Edition (WAIS IV) and standardized scores were considered. The findings were compared with 45 healthy controls (mean age 33.9 ± 10.7 years, range 17–66, 29 females) and a previously studied group of 74 beta‐thalassaemia patients (mean age 34.5 ± 10.3, range 16–66, 45 females; 21 non‐transfusion‐dependent [NTDT], 53 transfusion‐dependent [TDT]).^2^ Most controls were recruited among healthy patients' relatives in order to minimize the effect of different socio‐economic backgrounds. For all subgroups, exclusion criteria were a history of concomitant neurological disease, neurosurgery and severe head trauma. The study was approved by our local Ethics Committee and informed consent was obtained from all participants.

The groups did not differ regarding age or sex; education was similar between HS patients (14.7 ± 2.3 years) and controls (15.1 ± 3.9 years) and was higher than subjects with thalassaemia (11.5 ± 2.9 years, *p* < 0.001). According to testing, the mean Full‐Scale Intelligence Quotient (FSIQ) was significantly lower in HS than in healthy controls (88.2 ± 17.7 vs. 98.3 ± 13.6; *p* = 0.005, Mann–Whitney) and slightly though non‐significantly better than beta‐thalassaemia patients (88.2 ± 17.7 vs. 81.5 ± 13.5; *p* = 0.099; see Figure 1; Table 1). Subgrouping beta‐thalassaemia patients according to their phenotype, cognitive performances of HS patients did not differ from those of NTDT patients (85.9 ± 11.9, *p* = 0.810) while the scores were higher than those of TDT patients (79.8 ± 13.8; *p* = 0.039) though the significance did not survive after correction for multiple comparisons.

**FIGURE 1:**
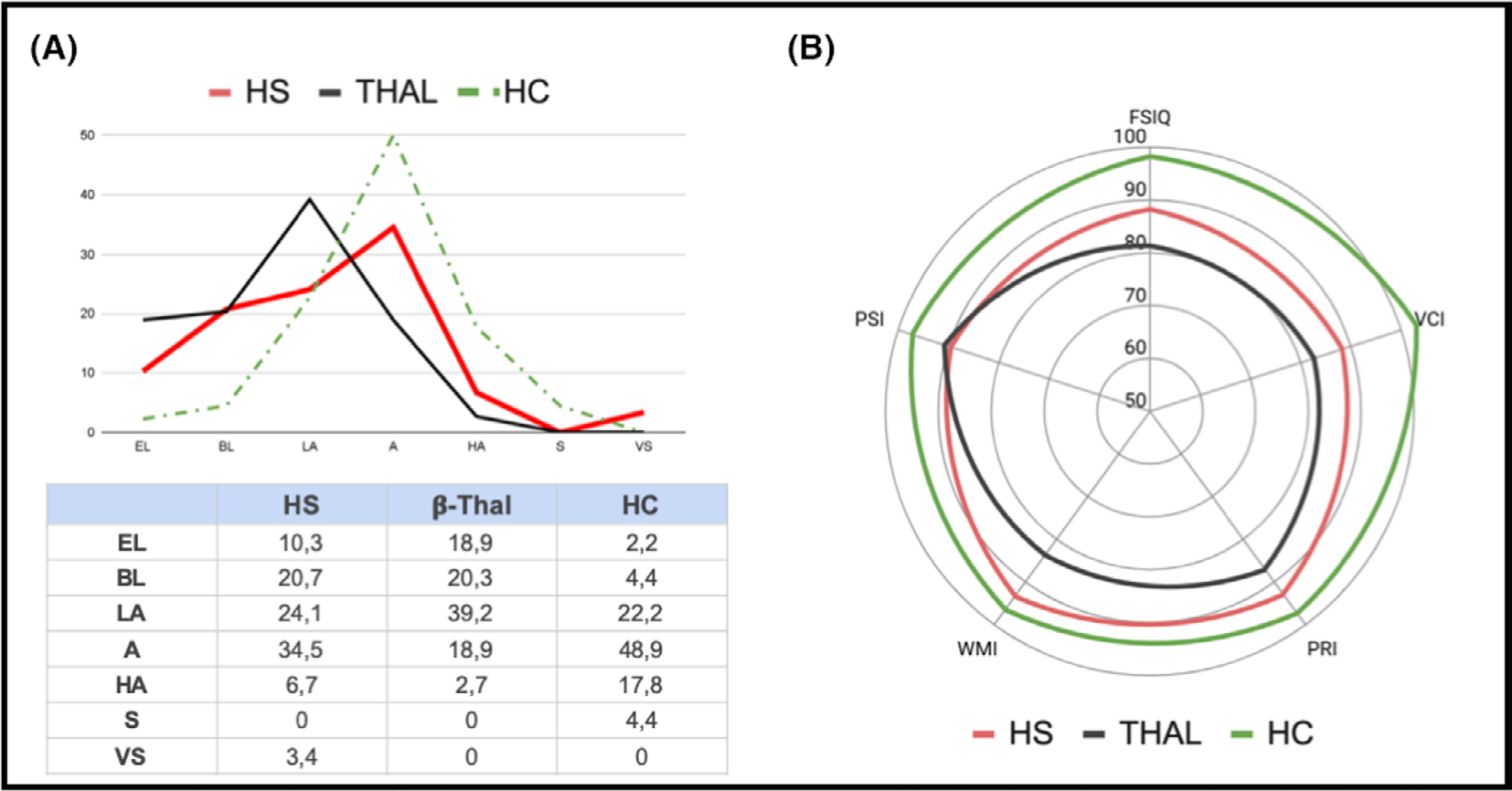
(A) Cognitive performances of hereditary spherocytosis (HS) patients according to Full‐Scale Intelligence Quotient (FSIQ) classes: Distribution among HS patients (HS, red line), beta‐thalassaemia patients (THAL; black line) and healthy controls (HC, dotted green line). The table indicate percentages; EL = extremely low (69 and below); BL = borderline (70–79); LA = low average (FSIQ 80–89); A = average (FSIQ 90–109); HA = high average (FSIQ 109–119); S = superior (FSIQ 120–129); VS = very superior (FSIQ 130 and above). (B) Radar plot of cognitive performances. Mean values and statistical analyses are reported in Table 1. PRI, Perceptual Reasoning Index; PSI, Processing Speed Index; VCI, Verbal Comprehension Index; WMI, Working Memory Index.

**TABLE 1:** Cognitive scores in hereditary spherocytosis, beta‐thalassaemia and healthy controls.

	HS	β‐THAL	HC	HS vs. THAL	HS vs. HC
	*M* ± SD	*M* ± SD	*M* ± SD	*p* (*U*, *r*)	*p* (*U*, *r*)
FSIQ	88.2 ± 17.7	81.5 ± 13.5	98.3 ± 13.6	*p* = 0.099 (1298, 0.16)	*p* **= 0.005** (**1941**, **0.33**)
VCI	88.1 ± 13.2	82.7 ± 15.4	103.0 ± 14.4	*p* = 0.072 (1319, 0.18)	*p* **< 0.001** (**281**, **0.48**)
PRI	92.9 ± 20.0	87.0 ± 12.3	97.4 ± 12.8	*p* = 0.189 (1253, 0.13)	*p* = 0.200 (537, 0.15)
WMI	93.3 ± 17.9	83.7 ± 15.2	96.4 ± 12.2	*p* **= 0.006** (**1446**, **0.27**)	*p* = 0.32 (564, 0.11)
PSI	89.5 ± 17.0	90.9 ± 15.1	97.3 ± 13.4	*p* = 0.612 (1005, 0.049)	*p* **= 0.033** ^a^ (**460**, **0.25**)

*Note*: *p*‐Values <0.05 are reported in bold. Complete testing findings are reported in Table S1. The VCI was obtained from the scores of similarities, vocabulary and information subtests, the PRI from the scores of block design, matrix reasoning and visual puzzles subtests, the WMI from the scores of digit span and arithmetic subtests, the PSI from the scores of symbol search and coding subtests. 95% confidence intervals for Mann–Whitney *U*‐test was [806; 1340] for HS versus THAL and [479; 829] for HS versus HC.

Abbreviations: FSIQ, Full‐Scale Intelligence Quotient; HC, healthy controls; HS, hereditary spherocytosis; PRI, Perceptual Reasoning Index; PSI, Processing Speed Index; *r*, effect size for Mann–Whitney test; *U*, *U* statistic for Mann–Whitney test; VCI, Verbal Comprehension Index; WMI, Working Memory Index; β‐THAL, beta‐thalassaemia.

^a^ Not significant after Bonferroni correction.

The FSIQ decrease in HS was mainly due to the low scores in the verbal comprehension (88.1 vs. 103, *p* < 0.001) and processing speed indices (PSI 89.5 vs. 97.3, *p* = 0.03) (see radar plot in Figure 1B; Table 1), while perceptual reasoning and working memory were less affected.

In HS, FSIQ scores did not correlate with the main laboratory findings; however, splenectomized HS patients had a trend of lower FSIQ scores than non‐splenectomized ones (85.1 ± 11.4 vs. 95.1 ± 19.4; *p* = 0.08).

In summary, the present pilot study first investigates cognitive functioning in HS patients showing the decreased cognitive performance of HS patients compared to healthy controls, particularly in the FSIQ and Verbal Comprehension Index (VCI) measures in spite of an equivalent education level. Even in the absence of severe anaemia, performances were similar to other chronic haemolytic anaemias (such as the non‐transfusion‐dependent beta‐thalassaemia subgroup). Among HS patients, splenectomy seems to further stratify cognitive performances suggesting that the main causative factor is not the lifelong red cell anomaly per se but rather the severity of anaemia and the related history of hospitalizations and treatments in the very early phases of life. Even though further research with larger samples is necessary, this study reveals a subgroup of HS patients (namely those with a history of splenectomy) that could benefit from cognitive support. Further genetic and neuroimaging analyses will help identify the structural and functional underpinnings of the preeminent impairment of the Verbal Domain that seems to represent a common feature in both adult HS and thalassaemia patients.

## AUTHOR CONTRIBUTIONS

RM and IT designed the study, analysed data, drafted the paper and had full access to all the data throughout the entire duration of the study. All authors collected data, reviewed and edited the draft and consented to the submission. RM and IT had full access to all the data throughout the entire duration of the study.

## FUNDING INFORMATION

None.

## CONFLICT OF INTEREST STATEMENT

Authors declare no conflict of interest.

## ETHICS APPROVAL STATEMENT

The study was approved by our local Ethics Committee (Prot. 0034213/i date 11/11/2022).

## PATIENT CONSENT STATEMENT

Informed consent was obtained by all participants.

## Supporting information


Table S1.

## Data Availability

The full set of data are available upon request to the corresponding author.
