# Supplementary material for: Cognitive impairment in hereditary spherocytosis
Source: Br J Haematol. 2025 Aug 15;207(4):1719–21. doi: 10.1111/bjh.70088 (PMC12512074; doi:10.1111/bjh.70088)
Supplement: Supplementary file 1 — Table S1. [file BJH-207-1719-s001.docx]

e-Table I: Mean values of WAIS-IV testing in Hereditary Spherocytosis patients compared to beta-thalassemia patients and healthy controls.

|  | **HS** | *ß-***THAL** | **HC** | **HS vs** *ß-***THAL**  **Mann-Whitney** | **HS vs HC Mann-Whitney** |
| --- | --- | --- | --- | --- | --- |
|  | M±sd | M±sd | M±sd | p (U, r) | **p (U, r)** |
| **Similarities** | 8.8±3.0 | 8.2±3.2 | 10.8±3.0 | 0.358 (1198.5, 0.091) | **0.010 (421, 0.3)** |
| **Vocabulary** | 7.9±2.9 | 6.6±2.7 | 10.3±3.6 | **0.016 (1491.5, 0.24)** | **0.003 (387, 0.34)** |
| **Information** | 7.3±2.9 | 6.4±3.1 | 10.2±2.8 | 0.174 (1259.5, 0.14) | **<0.001 (266.5, 0.5)** |
| **Block Design** | 8.5±3.1 | 7.9±2.2 | 9.4±3.1 | 0.478 (1170.5, 0.071) | 0.219 (540.5, 0.14) |
| **Matrix Reasoning** | 9.6±3.5 | 7.8±2.7 | 9.3±2.3 | **0.009 (1432.5, 0.26)** | 0.522 (711, 0.075) |
| **Visual Puzzles** | 8.7±4.5 | 8.0±3.1 | 9.9±2.3 | 0.834 (1102, 0.021) | **0.040 (467, 0.24)** |
| **Symbol Search** | 7.4±3.4 | 9.2±3.7 | 9.3±2.9 | 0.050 (805, 0.19) | **0.016 (435.5, 0.28)** |
| **Digit Span** | 9.4±3.7 | 7.1±3.0 | 9.9±3.0 | **0.002 (1483, 0.3)** | 0.596 (604, 0.062) |
| **Arithmetic** | 8.6±4.1 | 7.1±3.0 | 8.8±2.6 | 0.060 (1329.5, 0.19) | 0.741 (622.5, 0.038) |
| **Coding** | 8.7±3.9 | 7.5±2.7 | 9.6±2.8 | 0.184 (1254.5, 0.13) | 0.222 (541.5, 0.14) |

*Legend: HS=Hereditary Spherocytosis; ß-THAL= beta-thalassemia; HC=healthy controls; U=U statistic for Mann-Whitney test;* *r=effect size; FSIQ= full scale intelligence quotient; VCI=Verbal Comprehension Index; PRI=Perceptual Reasoning Index; WMI=Working Memory Index; PSI=Processing Speed Index. 95% confidence intervals for Mann-Whitney U test was*  *[806;1340] for HS vs. THAL and [479;829] for HS vs HC; P-values <0.05 are reported in red-bold. After Bonferroni correction, only Vocabulary, Information and Digit span differences remained significant.*
